# Increased PD-L1 Restricts Liver Injury in Nonalcoholic Fatty Liver Disease

**DOI:** 10.1155/2022/5954437

**Published:** 2022-05-16

**Authors:** Gang Dong, Xiaoquan Huang, Rongxin Chen, Ling Wu, Siyu Jiang, Shiyao Chen

**Affiliations:** ^1^Department of Gastroenterology and Hepatology, Zhongshan Hospital, Fudan University, Shanghai 200032, China; ^2^Liver Cancer Institute, Zhongshan Hospital, Fudan University and Key Laboratory of Carcinogenesis and Cancer Invasion, Ministry of Education, Shanghai 200032, China; ^3^Endoscopy Center and Endoscopy Research Institute, Zhongshan Hospital, Fudan University, Shanghai 200032, China; ^4^Center of Evidence-Based Medicine, Fudan University, Shanghai 200032, China

## Abstract

PD-L1 is a critical checkpoint that protects tissues from autoimmune injury. Nevertheless, the role of PD-L1 in nonalcoholic fatty liver disease- (NAFLD-) induced liver damage is still unclear. In this study, we examined the role and mechanism of PD-L1 expression on NAFLD-induced liver damage *in vitro* and *in vivo.* PD-L1 expression in the livers from patients with NAFLD, and LO2 cells treated by FFA, was significantly increased. FFA triggers a large amount of ROS (generated from NOX4 and damaged mitochondria), promoting the ZNF24 expression and suppressing ZN24 sumoylation, both of which enhance the PD-L1 transcription and expression. The knockdown of PD-L1 increases CD8 + T cells' damage to FFA-treated LO2 cells, while its upregulation limits the liver injury in NAFLD models. Collectively, we demonstrate that FFA promotes PD-L1 expression through the ROS/ZNF24 pathway and suppresses UBE2I-mediated ZNF24 sumoylation to enhance its transcriptional activity of PD-L1. PD-L1 upregulation limits FFA-induced injury of hepatocytes *in vitro* and *in vivo*.

## 1. Introduction

NAFLD is a clinicopathological syndrome that affects people who drink little to no alcohol. It is characterized by excessive fat deposition in hepatocytes. The three most common types of NAFLD are simple fatty liver (SFL), nonalcoholic steatohepatitis (NASH), and related cirrhosis [1, 2]. NAFLD causes decompensated cirrhosis, hepatocellular carcinoma, and recurrence of liver transplantation and affects the progress of other chronic liver diseases [1, 2]. The liver injury seems to have an essential role in the development and prognosis of NAFLD; however, it still remains unclear how to effectively alleviate liver injury in NAFLD.

Previous study has reported a higher number of CD8+ T cells in the NAFLD patients' liver. CD8+ T cells can aggravate the liver injury and accelerate the progress of NAFLD [3]. Contrary, depletion of CD8 + T cells can reduce hepatic inflammation and mitigate the liver damage caused by high-fat diets in mice [3, 4]. In addition to depleting CD8+ T cells, suppressing the dysfunction of CD8+ T cells in the liver may also be an effective way to alleviate liver damage induced by NAFLD.

The PD-1/PD-L1 pathway, which conveys immunosuppressive cosignal, is recognized as the immune checkpoint that negatively regulates the immune response. PD-L1 is expressed by a variety of cell types, including hepatocytes. PD-1 expression in T cells increases followed activation. PD-L1 on the tumor cells can bind to PD-1 on the activated T cells, leading to the inhibition of the cytotoxic T cells, thus promoting tumor invasion [5]. A similar process can be used to protect the heart and lung from autoimmune injury in the lupus model [6] and suppress autoimmune kidney disease [7]. However, so far, few studies investigated the role of PD-L1 in NAFLD-induced liver injury.

In this study, we examined the role and mechanism of PD-L1 expression on NAFLD-induced liver damage *in vitro* and *in vivo*. PD-L1 is providing an essential negative regulatory checkpoint to restrict hepatocytes injury in NAFLD.

## 2. Materials and Methods

### 2.1. Antibodies and Reagents

The following antibodies were used for western blot analysis: anti-PD-L1 (cat.no. NBP1-76769; NOVUS), anti-*β*-actin (cat.no. ab8224; Abcam), anti-NOX4 (cat.no. ab109225; Abcam), anti-ZNF24 (cat.no. NBP1-82866; NOVUS), anti-UBE2I (cat.no. ab75854; Abcam), and anti-SUMO-1 (cat.no. ab32058; Abcam). Antibodies used for immunohistochemistry analysis were the following: anti-PD-L1 (cat.no. NBP1-76769; NOVUS), anti-NOX4 (cat.no. ab109225; Abcam), anti-ZNF24 (cat.no. NBP1-82866; NOVUS), and anti-CD8 (cat.no. ab237709; Abcam). Anti-UBE2I (cat.no. ab75854; Abcam), anti-SUMO-1 (cat.no. ab32058; Abcam), and anti-ZNF24 (cat.no. A303-091A; Thermo Fisher Scientific) were used for Immunoprecipitation (IP). Oleic acid, palmitic acid, Oil Red O, and MitoTEMPO were purchased from Sigma-Aldrich (St. Louis, MO). Reactive oxygen species (ROS) assay, Nacetyl-cysteine (NAC), JC-1-Mitochondrial Membrane Potential Assay Kit, and LDH Cytotoxicity Assay Kit were obtained from Beyotime Institute of Biotechnology (Jiangsu, China). Alanine aminotransferase (ALT) and aspartate transaminase (AST) assay kit were acquired from Nanjing Jiancheng Bioengineering Institute (Jiangsu, China).

### 2.2. Cell Culture and Treatments

Human hepatocyte cell line LO2 cells (Chinese Academy of Science cell bank, Shanghai, China) were cultured in Dulbecco's modified Eagle's medium supplemented with 10% fetal bovine serum and 1% penicillin-streptomycin in a humidified cell incubator with 5% CO2 at 37°C. FFA stock solution was prepared as previously described [8]. A mixture of oleic acid and palmitic acid (OA : PA = 2 : 1) was used to culture cells. Peripheral blood sample (30 ml) was donated by healthy donors. Peripheral blood mononuclear cells (PBMCs) were obtained using Ficoll-Histopaque (Sigma, St. Louis MO) density centrifugation, as previously described [9]. Anti-CD8 microbeads packed in Miltenyi MidiMACS columns (Miltenyi Biotec, Auburn, CA) were used to purify CD8+ T cells from PBMCs according to the manufacturer's instructions. CD8+ T cells were then cultured in RPMI-1640 medium with 10% fetal bovine serum and stimulated with anti-CD3 and anti-CD28 monoclonal antibodies (BD Biosciences, CA, USA).

### 2.3. Liver Tissues

This study was approved by the Ethics Committee of Zhongshan Hospital of Fudan University (Shanghai, China). Normal and NAFLD livers were obtained from 10 patients who underwent medical treatment from 2018 to 2020. Clinical pathology data and follow-up of these patients were collected. The liver tissues were stored at -80°C.

### 2.4. Real-Time Reverse Transcription-PCR

Total RNA was extracted by TRIzol reagent (Invitrogen, USA) and then reverse-transcribed using a RevertAid First Strand cDNA Synthesis kit (Thermo Fisher Scientific, Inc.). The primers sequence is listed in Table 1. Real-time PCR was conducted using the FastStart Universal Probe Master (Roche, Basel, Switzerland). Target gene quantification was achieved by the equation of 2^-*ΔΔ*Ct^ and normalization using *β*-actin as the control.

### 2.5. Western Blot

Cells were lysed using RIPA buffer containing PMSF (Beyotime Institute of Biotechnology). Protein concentration was measured by BCA protein assay (Beyotime Institute of Biotechnology), protein samples (20 *μ*g/well) were then separated by 8%-12% SDS-PAGE, transferred onto a PVDF membrane (EMD Millipore), blocked by 5% low-fat milk, and incubated with primary antibodies at 4°C overnight. The next day, PVDF membranes were incubated with secondary antibodies (Beyotime Institute of Biotechnology) and analyzed by an enhanced chemiluminescence system (ECL, Pierce, Rockford, IL, USA).

### 2.6. Cell Transfection

siRNA against human NOX4, ZNF24, and PD-L1 and the appropriate scramble control siRNA were purchased from RiboBio Co., Ltd (Guangzhou, China). Plasmids overexpressing ZNF24 and the appropriate negative control were purchased from RiboBio Co., Ltd (Guangzhou, China). Lentivirus vectors overexpressing UBE2I, Sumo-1, and negative control were obtained from Genechem Co. Ltd (Shanghai, China). Briefly, LO2 cells (30-50% confluence for siRNA transfection; 50-80% confluence for plasmid transfection) were transfected with siRNA or plasmid in the presence of Lipofectamine 2000 (Invitrogen, Carlsbad, CA, USA) for 6 hours. LO2 cells were at 30%–50% confluence and transfected with lentivirus vectors at 5 *μ*g/mL polybrene for 24 h. Adeno-associated virus vectors expressing siRNA against rat PD-L1 and scramble control were obtained from Genechem Co. Ltd (Shanghai, China). Vectors were directly injected into the portal vein in rats.

### 2.7. Immunohistochemistry

Liver tissues were fixed with 4% paraformaldehyde before paraffin embedding and were then sectioned into 4 *μ*m thick slices. After deparaffinization, rehydration, and blocking with 10% BSA, these slices were incubated with primary antibodies at 4°C overnight. Next day, samples were then incubated with secondary antibodies. Diaminobenzidine (DAB) was chosen as the substrate chromogen. Hematoxylin and Eosin (H&E) were counterstained. Images were captured by a Leica microscope (Leica Microsystems, Germany).

### 2.8. Immunofluorescence

After being fixed with 4% paraformaldehyde, cells were permeabilized using 0.1% Triton X-100, blocked with 1% BSA, and incubated with primary antibodies at 4°C overnight. Next day, samples were then incubated with secondary antibodies. The nuclei were counterstained with DAPI. Images were captured by a Leica microscope (Leica Microsystems, Germany).

### 2.9. Co-Immunoprecipitation (co-IP)

Thermo Scientific IP Lysis Buffer (Cat. No. 87787) containing PMSF was used to extract proteins from cells. Primary antibodies were used to incubate protein samples on a shaker overnight at 4°C, followed by incubation with 100 *μ*l Protein A/G PLUS-Agarose beads (Cat. No. sc-2003; Santa Cruz Biotechnology, Inc.) for an additional 10 hours. Beads were then washed with Lysis Buffer 3 times and denatured using 2 × SDS sample buffer at 100°C for 20 min. The supernatant obtained by centrifugation was used for western blot analysis.

### 2.10. Dual-Luciferase Reporter Assay

LO2 cells were first transfected with various vectors overexpressing ZNF24, SUMO-1, or negative control, followed by transfection with reporter vectors of the pGL3-WT/MT-PD-L1 promoter or empty vector (Genomeditech Co. Ltd.). Forty-eight hours later, relative luciferase activity was analyzed by the Dual-Luciferase® Reporter Assay System (Promega, WI, USA), following the manufacturer's protocols.

### 2.11. Oil Red Staining

Staining of intracellular lipid accumulation was performed using Oil Red O (Sigma-Aldrich). After FFA treatment, LO2 cells were washed, fixed with 4% paraformaldehyde, stained with 0.35% Oil Red O solution, and washed three times with PBS, followed by visualization under the Leica microscope (Leica Microsystems, Germany) and photographed.

### 2.12. ROS

Intracellular ROS level was assessed by 2′,7′-Dichlorodihydrofluorescein diacetate (DCFH-DA) (Beyotime Institute of Biotechnology). After FFA treatment, LO2 cells were incubated with 10 *μ*M DCFH-DA for 30 min and washed using serum-free media. The Leica microscope (Leica Microsystems, Germany) was used to capture images.

### 2.13. LDH Measurement

The cytotoxicity of CD8+ T cells was assessed using the LDH Cytotoxicity Assay Kit (Beyotime Institute of Biotechnology) according to the manufacturer's instructions. FFA-treated LO2 cells (targets) were cocultured with CD8+ T cells (effectors) at an effector-to-target cell ratio of 50 : 1. Twelve hours later, the conditioned medium was collected and centrifuged. The obtained supernatant was subjected to LDH measurement using a spectrophotometer (Thermo Fisher Scientific) at 490 nm.

### 2.14. Mitochondrial Membrane Potential Assay

Mitochondrial membrane potential (*Δψ*m) was measured using JC-1 probes obtained from Beyotime Institute of Biotechnology (Jiangsu, China). LO2 cells after various treatments were incubated with JC-1 staining solution at 37°C for 20 min, followed by rinsing with staining buffer. The Leica microscope (Leica Microsystems, Germany) was used to capture the fluorescence intensity of both mitochondrial JC-1 monomers (green fluorescence) and aggregates (red fluorescence). The *Δψ*m was expressed as the ratio of red fluorescence to green fluorescence.

### 2.15. ALT/AST Assay

ALT and AST were determined using commercial assay kits according to the manufacturer's instructions.

### 2.16. Animal Experiments

Sprague–Dawley (SD) rats (250–280 g, 6–8 weeks) were obtained from Shanghai Slake Laboratory Animal Co., Ltd, China. All the animals were housed in an environment with a temperature of 22 ± 1°C, relative humidity of 50 ± 1%, and a light/dark cycle of 12/12 hr. All animal studies (including the mouse euthanasia procedure) were done in compliance with the regulations and guidelines of Zhongshan Hospital of Fudan University institutional animal care and conducted according to the AAALAC and the IACUC guidelines.

Rats were divided into two groups (*n* = 6 for each group): the control group fed with normal chow diets, and the NAFLD group fed with high-fat, high-fructose diets (HFHFD) (Trophic Animal Feed High-tech Co., Ltd. Nantong, China). Adeno-associated virus vectors expressing siRNA against rat PD-L1 and scramble control were directly injected into the portal vein in another SD rats (*n* = 6 for each group), all of which were fed with HFHFD diet. After 16 weeks, rats were euthanized, the liver tissues and blood were harvested for subsequent analysis. CD8+ T cells were isolated from the liver tissues using the MACS CD8 microbead kit (Miltenyi Biotech) following the manufacturers' instructions.

### 2.17. Statistical Analysis

Data analysis was carried out using SPSS 24.0 software (SPSS, Inc.) and indicated as means ± standard deviation. Student's unpaired *t*-test was used to compare two groups. More than two groups were analyzed by using One-Way ANOVA method. A *P* value < 0.05 was considered to be statistically significant.

## 3. Results

### 3.1. FFA Increases PD-L1 Expression in Hepatocytes

**Figure 1:**
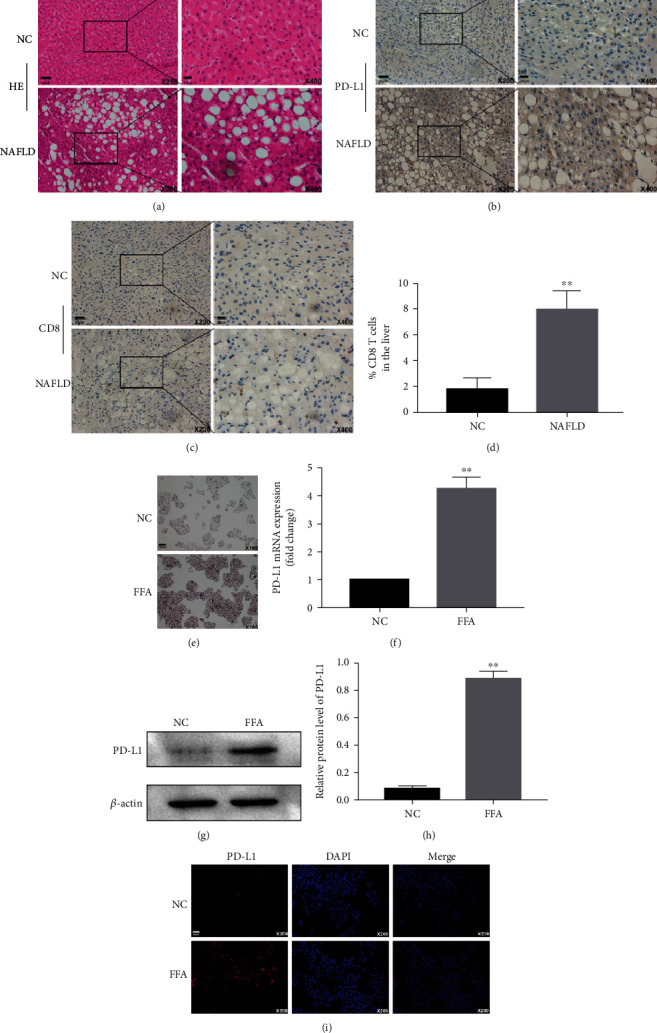
PD-L1 expression levels in liver samples and FFA-treated LO2 cells. (a–d) PD-L1 expression and CD8+ T cells in the liver tissues determined by immunohistochemistry. (e) Intracellular lipid accumulation after FFA treatment (0.8 mM) for 24 hour measured by Oil Red staining. (f–i) Expression levels of PD-L1 detected by qRT-PCR, western blot, and immunofluorescence. ^∗∗^*P* < 0.01.

As shown in Figures 1(a)–1(d), higher expression of PD-L1 was found in the liver of NAFLD patients compared to normal tissues. In addition, the number of CD8+ T cells in lesions was also increased. *In vitro*, FFA increased intracellular lipid deposition (Figure 1(e)) and enhanced PD-L1 mRNA expression in LO2 cells (Figure 1(f)), which was further confirmed by western blot (Figures 1(g) and 1(h)) or immunofluorescence (Figure 1(i)). Similar results were also found in primary hepatocytes (Figure S1). These results suggested that FFA promotes PD-L1 expression in hepatocytes.

### 3.2. ROS from NOX4 and Mitochondria Is Involved in the FFA-Induced PD-L1 Upregulation

**Figure 2:**
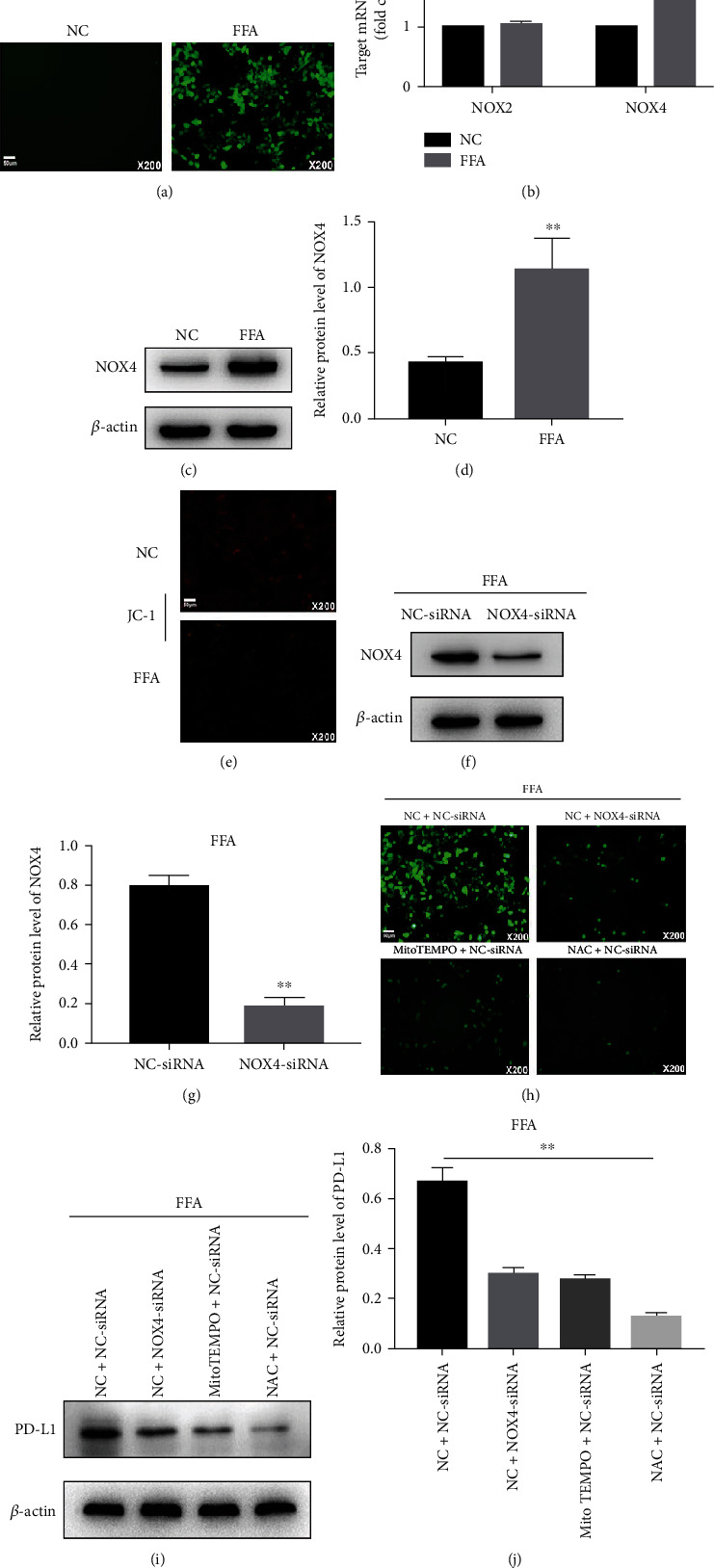
ROS mediated PD-L1 upregulation in FFA-treated LO2 cells. (a) Intracellular ROS in LO2 cells before and after FFA treatment measured by DCFH-DA. (b–d) Expression levels of NOX2 and NOX4 measured using qRT-PCR and western blot. (e) JC-1 probes were used to detect mitochondrial membrane potential. (f–j) Intracellular ROS in LO2 cells pretreated with siRNA against NOX4, MitoTEMPO (10 *μ*M), or NAC (5 mM) followed by FFA treatment, measured by DCFH-DA, PD-L1 expression determined by western blot. ^∗∗^*P* < 0.01.

As shown in Figure 2(a), in LO2 cells treated with FFA, ROS levels were significantly increased. Mitochondria and nicotinamide adenine dinucleotide phosphate oxidase (NOX) have been reported to be the major sources of ROS [10, 11]. FFA promoted the NOX4 expression (other NOX family members were not detected) and decreased the mitochondrial membrane potential (Figures 2(b)–2(e)). Knockdown of NOX4 or application of the mitochondrion-targeted antioxidant (MitoTEMPO) or NAC reduced ROS levels (Figures 2(f)–2(h)), and, in turn, decreased the expression of PD-L1 (Figures 2(i) and 2(j)). These results indicate that ROS from NOX4 and damaged mitochondria promotes PD-L1 expression in LO2 cells treated with FFA.

### 3.3. ZNF24-Mediated ROS Induces PD-L1 Expression

**Figure 3:**
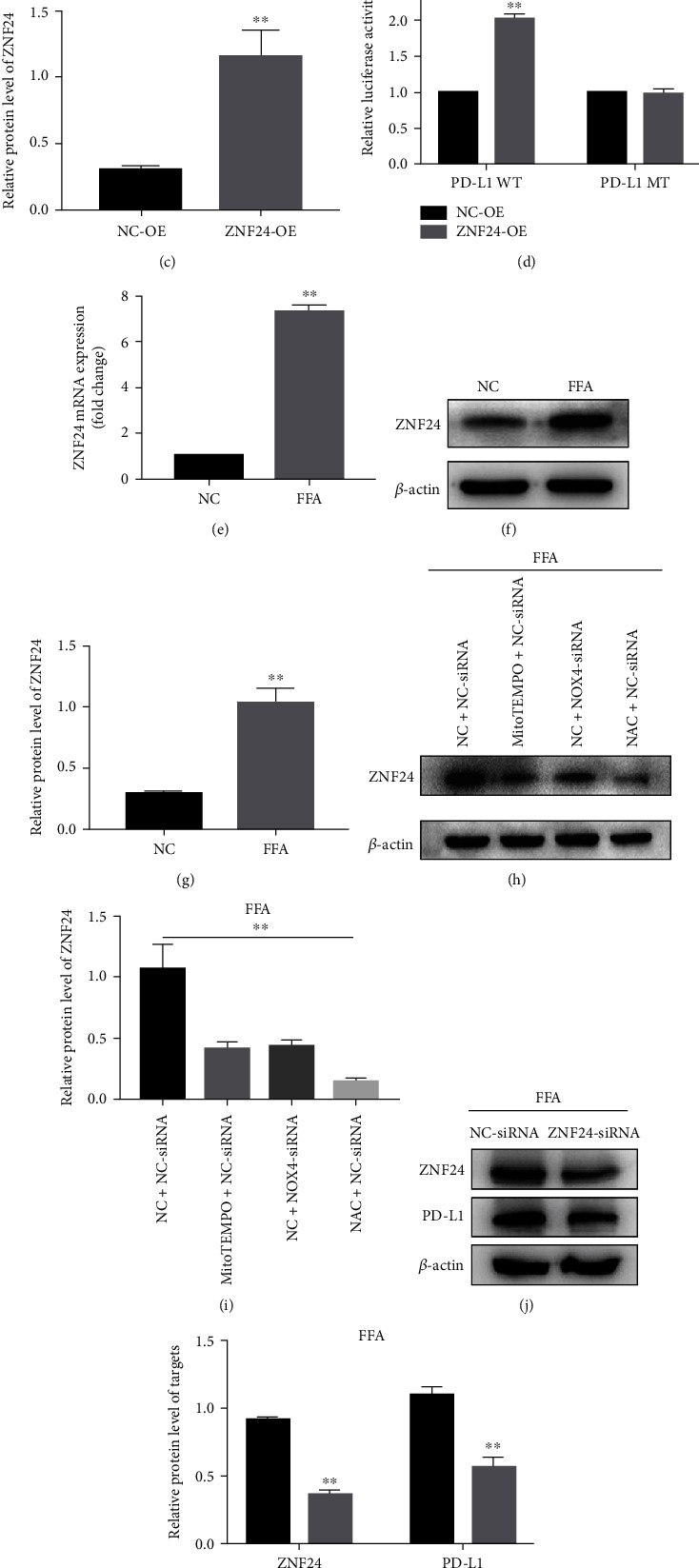
ZNF24 promoted PD-L1 expression through binding to its promoter in FFA-treated LO2 cells. (a) A schematic of the target sites (wild and mutant) of ZNF24 in the promoter of PD-L1. (b–d) Dual-luciferase reporter assays performed in LO2 cells transfected with WT or MT plasmid containing ZNF24-binding sites in the PD-L1 promoter using Lipofectamine 2000 after ZNF24 overexpression. (e–g) ZNF24 expression after FFA treatment determined by qRT-PCR and western blot. (h and i) ZNF24 expression in LO2 cells pretreated with siRNA against NOX4, MitoTEMPO (10 *μ*M), or NAC (5 mM) followed by FFA treatment, measured by western blot. (j and k) ZNF24 and PD-L1 expression in LO2 cells pretreated with siRNA against ZNF24, followed by FFA treatment detected by western blot. ^∗∗^*P* < 0.01.

Using the website: http://jaspar.genereg.net/, we found one ZNF24-binding site in the promoter region of PD-L1 (Figure 3(a)). ZNF24 binds to the PD-L1 promoter through the aforementioned sites and promotes PD-L1 transcription (Figures 3(b)–3(d)). Moreover, FFA increases ZNF24 expression (Figures 3(e)–3(g)). Knockdown of NOX4 or application of MitoTEMPO or NAC inhibited ZNF24 expression (Figures 3(h)–3(i)). PD-L1 expression was suppressed following the ZNF24 knockdown (Figures 3(j)–3(k)). These results show that ZNF24-mediated ROS induces the expression of PD-L1.

### 3.4. ZNF24 Sumoylation Mediated by UBE2I Restrains the Promotion of ZNF24 on PD-L1 Transcription

**Figure 4:**
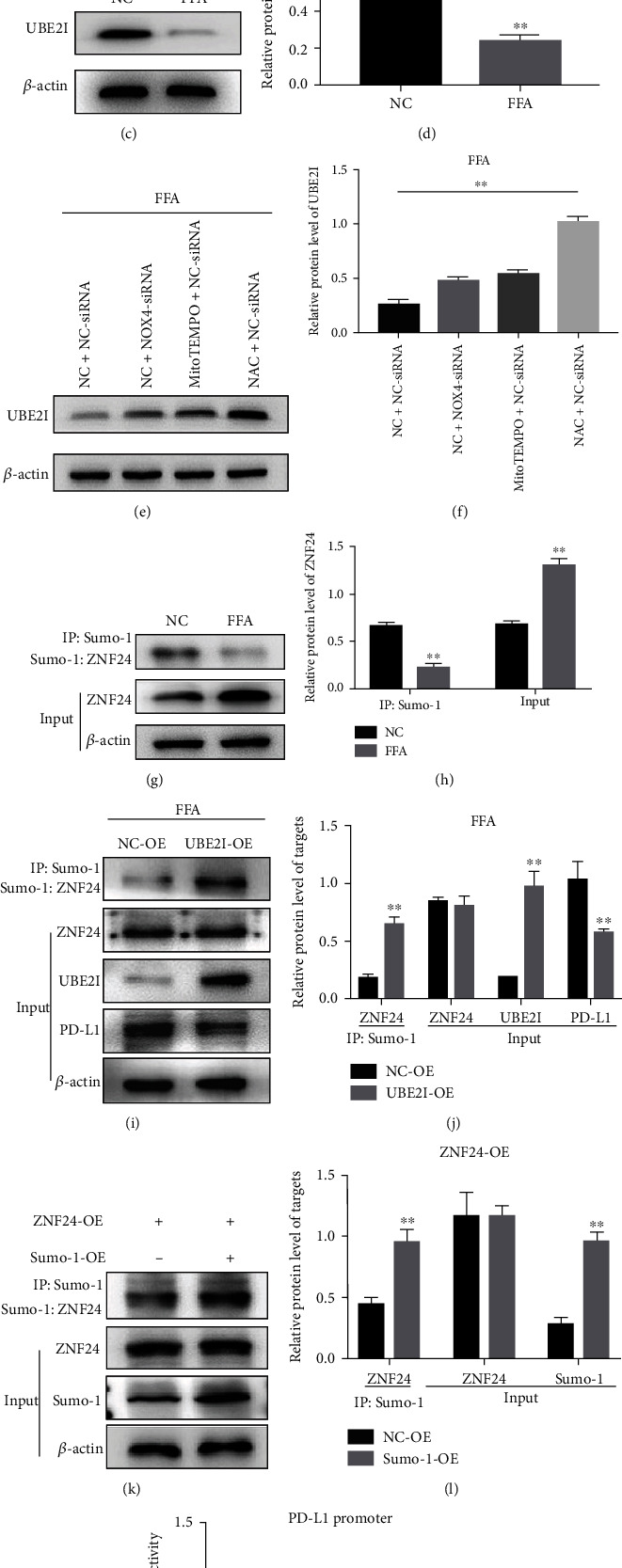
Identification of ZNF24–UBE2I protein interactions. (a and b) Analysis of protein interactions between ZNF24 and UBE2I using the BioGRID database, further confirmed by Co-IP assays. (c–f) UBE2I expression in LO2 cells treated by FFA or pretreated with siRNA against NOX4, MitoTEMPO (10 *μ*M), or NAC (5 mM) followed by FFA treatment measured by western blot. (g and h) After FFA treatment, Sumo-1: ZNF24 and ZNF24 expression levels detected by western blot. (i and j) After UBE2I overexpression, Sumo-1: ZNF24, ZNF24, PD-L1, and UBE2I expression levels were determined by western blot. (k–m) Dual-luciferase reporter assays performed in LO2 cells transfected with WT plasmid containing ZNF24-binding sites in the PD-L1 promoter using Lipofectamine 2000 after ZNF24 overexpression with or without Sumo-1 overexpression. ^∗∗^*P* < 0.01.

As shown in Figure 4(a), an interaction between ZNF24 and UBE2I was observed using the protein interaction database. This interaction was further confirmed using CoIP assays (Figure 4(b)). FFA inhibited UBE2I expression, which was reversed by NOX4 knockdown or application of MitoTEMPO or NAC (Figures 4(c)–4(f)); ZNF24 sumoylation was also suppressed after FAA treatment (anti-SUMO-2/3 antibody did not pull down ZNF24, data not shown) (Figures 4(g) and 4(h)). However, overexpression of UBE2I increased ZNF24 sumoylation and decreased PD-L1 expression in FFA-treated LO2 cells (Figures 4(i) and 4(j)). Moreover, overexpressing ZNF24 and SUMO-1 at the same time enhanced the degree of sumoylation of ZNF24 (Figures 4(k) and 4(l)) but restrained the promotive effects of ZNF24 on PD-L1 transcription (Figure 4(m)). These data suggest that FFA can inhibit the expression of UBE2I, which mediates the sumoylation of ZNF24 and then suppresses its transcriptional activity of PD-L1.

### 3.5. Knockdown of PD-L1 Increases the Damage of CD8 + T Cells to FFA-Treated LO2 Cells

**Figure 5:**
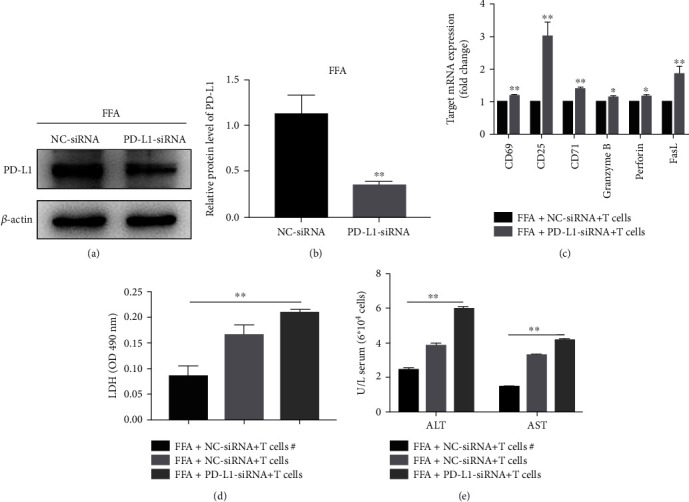
PD-L1 knockdown aggravated the damage of CD8 + T cells to FFA-treated LO2 cells. (a and b) Western blot determined the effect of siRNA against PD-L1. Coculturing of FFA-treated LO2 cells and CD8 + T cells *in vitro*. (c) mRNA expression levels of markers of T cell activation measured by qRT-PCR, (d) LO2 cell injury was evaluated by LDH assay, (e) AST or ALT in the supernatants measured by commercial assay kits. ^#^ represents that CD8 + T cells were incubated separately with LO2 cells, but supernatants were put together. ^∗^*P* < 0.05, ^∗∗^*P* < 0.01.

Coculturing of FFA-treated LO2 cells and CD8 + T cells *in vitro* was used to evaluate the cytotoxicity of CD8 + T cells to LO2 cells. As shown in Figure 5, after knockdown of PD-L1, the activation of CD8 + T cells increased, and the damage to LO2 cells was aggravated.

### 3.6. PD-L1 Limits Liver Injury in a NAFLD Rat Model

**Figure 6:**
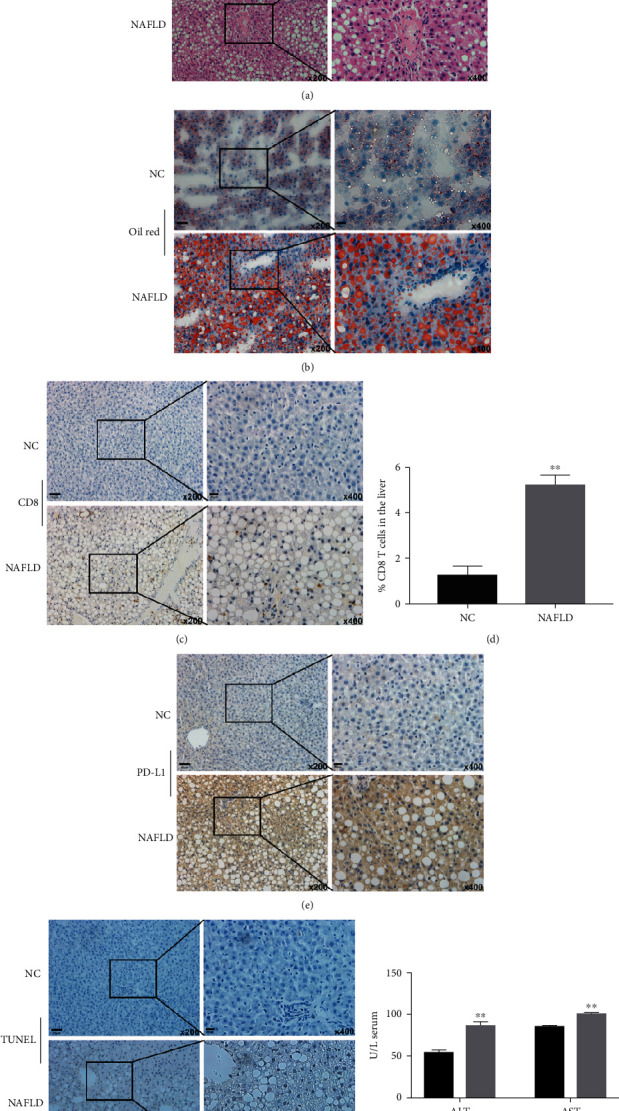
A NAFLD model was established. (a and b) The pathological changes of NAFLD were verified by H&E and Oil Red staining. (c–f) CD8+ T cells, PD-L1 expression, and hepatocyte apoptosis were detected by immunohistochemistry. (g) ALT and AST in the serum were detected using the commercial kit. ^∗∗^*P* < 0.01.

**Figure 7:**
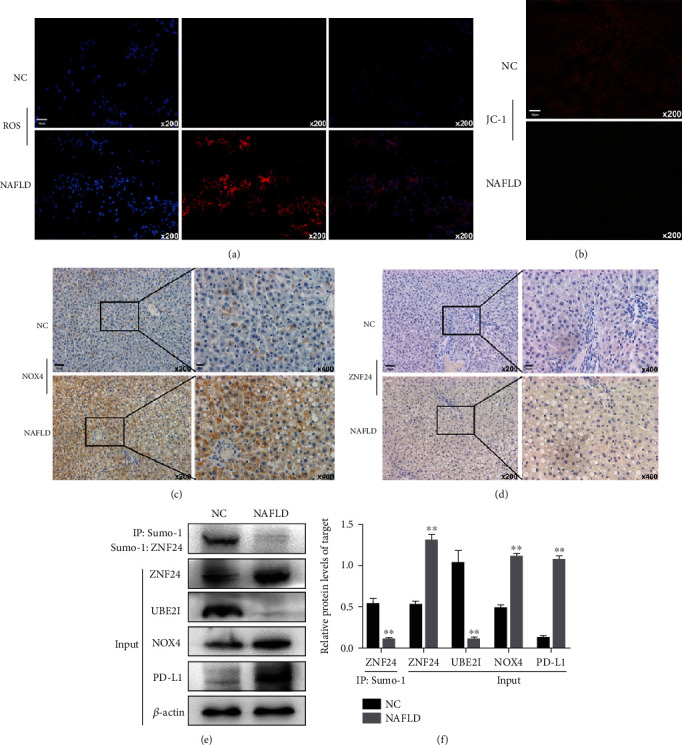
ROS/ZNF24/PD-L1 pathway activation in NAFLD models. (a) Intracellular ROS in hepatocytes in NAFLD significantly increased. (b) JC-1 probes were used to detect mitochondrial membrane potential. (c and d) NOX4 and ZNF24 expressions were determined by immunohistochemistry. (e and f) ROS/ZNF24/PD-L1 pathway activation examined by western blot.

**Figure 8:**
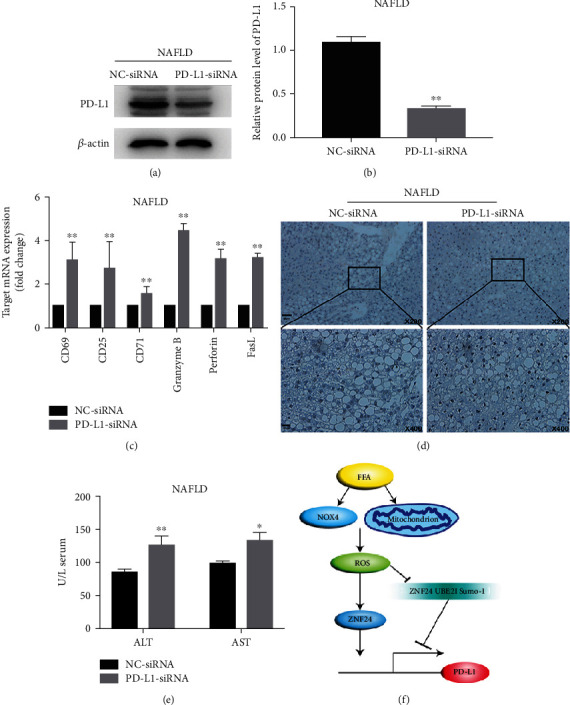
PD-L1 limits liver injury in NAFLD models. (a and b) Western blot determined the effect of siRNA against PD-L1. (c) After PD-L1 knockdown, mRNA expression levels of markers of T cell activation measured by qRT-PCR. (d and e) Hepatocyte injury was evaluated by the Tunel assay and ALT/AST measurement. (f) Graph illustrating that both ROS/ZNF24 pathway activation and UBE2I-mediated ZNF24 sumoylation suppression induced by FFA promoted PD-L1 expression. ^∗^*P* < 0.05, ^∗∗^*P* < 0.01.

In order to observe the effect of PD-L1 on NAFLD- (rats fed with high-fat, high-fructose diet) induced liver injury *in vivo*, we established a NAFLD model (Figure S2). As shown in Figures 6(a) and 6(b), a large amount of lipid accumulated in hepatocytes, an accumulation of CD8 + T cells in NAFLD (Figures 6(c) and 6(d)), and increased expression of PD-L1 expression, hepatocytes apoptosis, and the level of ALT and AST were observed in the NAFLD model (Figures 6(e)–6(g)). In addition, large amounts of ROS were found (Figures 7(a)–7(d)), accompanied by mitochondrial damage, NOX4 and ZNF24 upregulation. The above results were further confirmed by western blot (Figures 7(e) and 7(f)). After knockdown of PD-L1, the activation of CD8 + T cells increased, and the damage to hepatocytes was aggravated (Figure 8). These results suggest that increased PD-L1 expression limits liver injury in NAFLD models.

## 4. Discussion

As a clinicopathological syndrome, nonalcoholic fatty liver disease (NAFLD) is one of the most common chronic liver diseases worldwide. Its prevalence ranges from 6 to 35% in different countries [1, 2, 12, 13]. NAFLD may appear as simple steatosis to hepatocellular injury, inflammation, and cirrhosis, which are the leading cause of end-stage liver disease, such as hepatocellular carcinoma. One of the key ways to prevent NAFLD from developing into the end-stage liver disease is to effectively restrict hepatocyte injury [1, 2, 12, 13]. Although it has attracted global attention at present, the pathological mechanism of liver injury in NAFLD remains largely unclear.

A previous study found an accumulation of CD8 + T cells in the livers of NAFLD patients. CD8+ T cells trigger NAFLD-induced liver damage and drive the progression of NAFLD. CD8 + T cells depletion can significantly reduce hepatic inflammation and alleviate the liver damage caused by high-fat diets in mice [3, 4]. Therapies targeting CD8 + T cells may be a novel treatment for NAFLD. Besides the depletion of CD8 + T cells, it is necessary to investigate whether there are any simple but effective ways to suppress the dysfunction of CD8 + T cells, thus retarding the development of NAFLD.

PD-L1 can suppress the immune response by binding with PD-1 on the surface of T lymphocytes. This mechanism is used by tumor cells to escape from T cell-mediated cytotoxicity [14]. Several drugs targeting the PD1/PD-L1 pathway have been used in clinical malignant tumor patients, resulting in a favorable prognosis [15, 16]. In addition, PD-L1 has also been reported to be associated with several autoimmune diseases such as Graves' disease [17, 18], lupus [6], and type 1 diabetes [19]. For example, PD-L1 was a critical checkpoint that protected the heart and lung from autoimmune injury in the lupus models. The blockade of the PD-1/PD-L1 pathway accelerates autoimmune disease [6]. In the sepsis model, restoring PD-L1 expression improves mouse survival and alleviates liver injury [20]. However, up to this date, the role of PD-L1 in NAFLD-induced liver damage remains poorly understood. In this study, we hypothesized that PD-L1 might be involved in liver injury in NAFLD. PD-L1 upregulation may have an important role in limiting liver damage. We observed that PD-L1 expression levels in the livers from NAFLD were higher compared to that from a normal subject and in the NAFLD cell model induced by FFA. In particular, the total expression of PD-L1 on the surface of hepatocytes is higher than that of other cell types in the liver (Figure S3).

Next, we investigated the mechanism of FFA to promote PD-L1 upregulation in hepatocytes. Previous studies reported that FFA could increase the level of intracellular ROS [21]. The major source of ROS is mitochondria and NADPH oxidase (NOX) [10, 11]. Our data indicated that FFA can promote intracellular ROS generation; both mitochondria and NOX4 were involved in ROS production. Application of MitoTEMPO, NAC, or siRNA against NOX4 can decrease ROS levels. ROS inducers were reported to increase the expression of PD-L1 in tumor-associated macrophages (TAMs), ROS scavenging repressed PD-L1 expression [22]. Consistent with this report, we found that the administration of siRNA against NOX4, MitoTEMPO, or NAC decreased ROS levels and reversed the PD-L1 upregulation induced by FFA.

ZNF24, also known as ZNF191, belongs to the SCAN domain subfamily of the Krüppel-like zinc finger transcription factors. It possesses the transrepression activity of the GAL4 promoter in NIH-3 T3 cells and can bind to the *β*-catenin promoter [23, 24]. However, little is known on its role in FFA-induced PD-L1 upregulation. In this study, we found that ZNF24 has one binding site in the PD-L1 promoter region, which was confirmed by luciferase and CHIP assay. Similar to PD-L1, FFA increased ZNF24 expression and regulated by ROS. Therefore, ROS/ZNF24 pathway is considered to participate in the PD-L1 upregulation induced by FFA. In the present study, an interaction between ZNF24 and UBE2I was also observed using the protein interaction database and was corroborated using co-IP assays. UBE2I, a homolog of the E2 ubiquitin-conjugating enzyme, is involved in the covalent linking of the SUMO-1 molecule to target proteins and regulate their sumoylation [25]. UBE2I mediated ZNF24 sumoylation and suppressed ZNF24 transcriptional activity, decreasing PD-L1 expression in FFA-treated hepatocytes.

To further investigate the *in vivo* consequences of PD-L1 expression, we constructed a NAFLD rat model based on previous reports [26]. Our results revealed that ROS/ZNF24 pathway was activated, and the PD-L1 expression was upregulated. It may be because FFA itself can directly damage hepatocytes [27], so, there was a certain degree of apoptosis in hepatocytes, and serum transaminase was increased; however, the liver injury was aggravated, followed by PD-L1 knockdown (Figures 8(d) and 8(e)) or application of PD-1, but the liver injury was relieved after the use of NOX4 inhibitors (Figure S4). Although increased PD-L1 expression limited NAFLD-induced liver injury, this still did not clarify whether it was appropriate to select PD-L1 related targeted drugs for NAFLD patients who have liver cancer, and if yes, how to administer these drugs. All of these need to be further explored by subsequent experiments.

In conclusion, the study revealed that FFA promotes PD-L1 expression through the ROS/ZNF24 pathway and suppresses UBE2I-mediated ZNF24 sumoylation, thus enhancing its transcriptional activity. PD-L1 upregulation limited FFA-induced injury of hepatocytes *in vitro* and *in vivo*.

## Figures and Tables

**Table 1:** Primers.

Gene	Sense	Antisense
Human		
PD-L1	**GACCACCACCACCAATTCCAAGAG**	**TGGAGGATGTGCCAGAGGTAGTTC**
NOX2	**TTCCAGTGCGTGCTGCTCAAC**	**TGGTGTGAATCGCAGAGTGAAGTG**
NOX4	**GTGTCTAAGCAGAGCCTCAGCATC**	**CGGAGGTAAGCCAAGAGTGTTCG**
ZNF24	**TTGTTGCCATCCTACCCAAAGAGC**	**CTCCAAATCCTCCAGCACTGTCAC**
CD69	**GTCCTTCCAAGTTCCTGTCCTGTG**	**ACATGGCTGTCTGATGGCATTGAG**
CD25	**GCTCTGCCACTCGGAACACAAC**	**AGGCTCGCTTGGTCCACTGG**
CD71	**TGAGGGAGGAGCCAGGAGAGG**	**CTTGATGGTGCCGGTGAAGTCTG**
Granzyme B	**GTGCGGTGGCTTCCTGATACG**	**TGCTGGGTCGGCTCCTGTTC**
Perforin	**GGCATCCACGGCAGCATCTC**	**CAGCAGGTCGTTAATGGAGGTGTG**
FasL	**ACCGCCACCACTACCACCTC**	**CCTACCAAGGCAACCAGAACCATG**
Rat		
CD69	**GAGAGAGGGCAGAGGGACCATG**	**GACCACTACGAGCACAGCACAAG**
CD25	**ACCACGGACACGCAGAAATCAAC**	**AGGAAGCCTCACTCTCTGGGAAAG**
CD71	**AGTGATGCCTGAAGCCTCCTCTC**	**GTCGCTGAACTTTGCATTGCTGAG**
Granzyme B	**GACATGAAGCCAAGCCCCACTC**	**CTCGTATAAGGAAGCCGCCACAC**
Perforin	**GTCTCGGTCCTCACGGCTCTG**	**ACGCTCAAGCAGTCTCCTACCTC**
FasL	**TCACCACTACCACCGCCTTCC**	**CCATTCCAACCAGAGCCACCAG**

## Data Availability

All datasets analyzed for this study are included in the article material.

## Abbreviations

NAFLD: Nonalcoholic fatty liver disease
ROS: Reactive oxygen species
SFL: Simple fatty liver
NASH: Nonalcoholic steatohepatitis
NAC: Nacetyl-cysteine
ALT: Alanine aminotransferase
AST: Aspartate transaminase
OA and PA: Oleic acid and palmitic acid
PBMCs: Peripheral blood mononuclear cells
DAB: Diaminobenzidine
H&E: Hematoxylin and eosin
Co-IP: Co-immunoprecipitation
DCFH-DA: Dichlorodihydrofluorescein diacetate
SD: Sprague–Dawley
HFHFD: High-fat, high-fructose diets
NOX: Nicotinamide adenine dinucleotide phosphate oxidase
TAMs: Tumor-associated macrophages.

## Supplementary Materials

Supplementary Materials Figure S1: PD-L1 expression levels in FFA-treated primary hepatocytes. (A-B) Rat primary hepatocytes were isolated and identified by using CK18 antibody. (C-D) PD-L1 expression levels were analyzed by qRT-PCR and western blot. Figure S2: the rat NASH model was established. (A-B) Liver triglyceride and serum insulin levels were detected. (C) Fibrotic deposition was determined by Masson staining. Figure S3: PD-L1 (red) and cell-specific markers (CD3, CD19, CD68, and CK18) (green) were counterstained in the liver sections. Red and green merged into yellow. Figure S4: hepatocyte injury was detected after intervening with PD-1 antibody or NOX4 inhibitor in the rat NASH model. Hepatocyte injury was evaluated by the Tunel assay (A) and ALT/AST measurement (B-C). Table 1: primers used for qRT-PCR analysis.
