# Supplementary material for: Increased PD-L1 Restricts Liver Injury in Nonalcoholic Fatty Liver Disease
Source: Oxid Med Cell Longev. 2022 May 16;2022:5954437. doi: 10.1155/2022/5954437 (PMC9126662; doi:10.1155/2022/5954437)
Supplement: Supplementary Materials — Figure S1: PD-L1 expression levels in FFA-treated primary hepatocytes. (A-B) Rat primary hepatocytes were isolated and identified by using CK18 antibody. (C-D) PD-L1 expression levels were analyzed by qRT-PCR and western blot. Figure S2: the rat NASH model was established. (A-B) Liver triglyceride and serum insulin levels were detected. (C) Fibrotic deposition was determined by Masson staining. Figure S3: PD-L1 (red) and cell-specific markers (CD3, CD19, CD68, and CK18) (green) were counterstained in the liver sections. Red and green merged into yellow. Figure S4: hepatocyte injury was detected after intervening with PD-1 antibody or NOX4 inhibitor in the rat NASH model. Hepatocyte injury was evaluated by the Tunel assay (A) and ALT/AST measurement (B-C). Table 1: primers used for qRT-PCR analysis. [file 5954437.f1.zip › Table S1.docx]

**Table 1 Primers.**

**Human**

| **Gene sense** | **anti-sense** |
| --- | --- |
| **PD-L1 GACCACCACCACCAATTCCAAGAG** | **TGGAGGATGTGCCAGAGGTAGTTC** |
| **NOX2 TTCCAGTGCGTGCTGCTCAAC** | **TGGTGTGAATCGCAGAGTGAAGTG** |
| **NOX4 GTGTCTAAGCAGAGCCTCAGCATC** | **CGGAGGTAAGCCAAGAGTGTTCG** |
| **ZNF24 TTGTTGCCATCCTACCCAAAGAGC** | **CTCCAAATCCTCCAGCACTGTCAC** |
| **CD69 GTCCTTCCAAGTTCCTGTCCTGTG** | **ACATGGCTGTCTGATGGCATTGAG** |
| **CD25 GCTCTGCCACTCGGAACACAAC** | **AGGCTCGCTTGGTCCACTGG** |
| **CD71 TGAGGGAGGAGCCAGGAGAGG** | **CTTGATGGTGCCGGTGAAGTCTG** |
| **Granzyme B GTGCGGTGGCTTCCTGATACG** | **TGCTGGGTCGGCTCCTGTTC** |
| **Perforin GGCATCCACGGCAGCATCTC** | **CAGCAGGTCGTTAATGGAGGTGTG** |
| **FasL ACCGCCACCACTACCACCTC** | **CCTACCAAGGCAACCAGAACCATG** |

**Rat**

| **CD69 GAGAGAGGGCAGAGGGACCATG** | **GACCACTACGAGCACAGCACAAG** |
| --- | --- |
| **CD25 ACCACGGACACGCAGAAATCAAC** | **AGGAAGCCTCACTCTCTGGGAAAG** |
| **CD71 AGTGATGCCTGAAGCCTCCTCTC** | **GTCGCTGAACTTTGCATTGCTGAG** |
| **Granzyme B GACATGAAGCCAAGCCCCACTC** | **CTCGTATAAGGAAGCCGCCACAC** |
| **Perforin GTCTCGGTCCTCACGGCTCTG** | **ACGCTCAAGCAGTCTCCTACCTC** |
| **FasL TCACCACTACCACCGCCTTCC** | **CCATTCCAACCAGAGCCACCAG** |
